# Nuclear organization and regulation of the differentiated state

**DOI:** 10.1007/s00018-020-03731-4

**Published:** 2021-01-28

**Authors:** Eliya Bitman-Lotan, Amir Orian

**Affiliations:** Rappaport Research Institute and Faculty of Medicine, The Rappaport Faculty of Medicine Technion-IIT, Technion Integrative Cancer Center (TICC), Technion-Israel Institute of Technology, Bat-Galim, 3109610 Haifa, Israel

**Keywords:** Cell identity, Aging, Gene regulation, Lamin, Chromatin, H3K9me3, LLPS

## Abstract

Regulation of the differentiated identity requires active and continued supervision. Inability to maintain the differentiated state is a hallmark of aging and aging-related disease. To maintain cellular identity, a network of nuclear regulators is devoted to silencing previous and non-relevant gene programs. This network involves transcription factors, epigenetic regulators, and the localization of silent genes to heterochromatin. Together, identity supervisors mold and maintain the unique nuclear environment of the differentiated cell. This review describes recent discoveries regarding mechanisms and regulators that supervise the differentiated identity and protect from de-differentiation, tumorigenesis, and attenuate forced somatic cell reprograming. The review focuses on mechanisms involved in H3K9me3-decorated heterochromatin and the importance of nuclear lamins in cell identity. We outline how the biophysical properties of these factors are involved in self-compartmentalization of heterochromatin and cell identity. Finally, we discuss the relevance of these regulators to aging and age-related disease.

## Introduction: a short historic perspective

How differentiated cells maintain their identity is a fundamental question that researchers have been grappling with since the early days of modern biology. The “textbook view” was that the differentiated identity of cells is determined by sequential steps, and the term “terminal differentiation” was coined as a sign of an irreversible process (for reviews describing mechanisms of terminal differentiation see [1, 2]). It is now clear, however, that the differentiated state is plastic, enabling re-programming, de-differentiation, and trans-differentiation [3].

What maintains differentiated cell identity? Conard Waddington once compared differentiation to balls rolling downhill, where the balls (i.e. the differentiating cells) are funneled into specific valleys of irreversible fates [4, 5]. A large body of evidence suggests, however, that above the Waddington valleys is a molecular safety net that prevents the differentiated cells from wandering off into other valleys [6–8]. Identity supervision serves as a barrier, inhibiting spontaneous reprograming or trans-differentiation as well as tumorigenesis; its loss is a hallmark of aging, and results in metabolic disorders, such as diabetes, neurodegeneration, and cancer. Manipulation of the activity of central nodes within identity networks will enable better and more efficient reprogramming, which can be of high relevance to regenerative medicine, aging biology, and cancer therapy/treatment.

The nucleus plays a central role in identity regulation. Sir J. Gurdon, who conducted nuclear transfer experiments as early as 1962, described the generation of adult frogs by transplanting a single nucleus from a somatic cell into a fertilized egg whose nucleus was removed [9, 10]. Later on, the Weintraub laboratory forced expression of transcription factor MyoD in differentiated fibroblasts resulting in conversion to muscle-like cells and establishing a role for transcriptional regulation of cell identity [11]. In those exciting early days of myogenic differentiation, Blau and Baltimore postulated that both passive and active chromatin-related mechanisms within the cell nucleus are required for maintaining the differentiated identity [12]. Indeed, returning to those pioneering experiments using global genome-wide studies, it was demonstrated that MyoD-induced trans-differentiation is accompanied by re-wiring and remodeling the 3D of the genome, forcing the expression of myogenic programs [13]. Over the years, additional transcription factors have been shown to be required to maintain cell identity, including Pax5, a homeobox transcription factor that is required for B cell fate and identity. Elimination of Pax5 in fully differentiated B cells resulted in their trans-differentiation to immature T cells and in the development of T cell lymphomas [14–17]. Moreover, Yamanaka and colleagues discovered that expression of four transcription factors (Oct4, Sox2, Klf4, c-Myc; OSKM) induced reprogramming of fibroblasts to form pluripotent cells (iPS), a discovery that was awarded the 2012 Nobel prize in physiology and medicine [18].

In addition to sequence-specific transcription factors, the regulation of large-scale chromatin and nuclear organization is emerging as crucial for regulation of cell identity. Recent findings, presented below, suggest that cell identity is maintained at the level of high-order chromatin and nuclear organization involving both genetic and epigenetic players as well as biophysical forces that safeguard cell identity. The changes in cell identity supervision during aging and the relevance of these perturbations to aged-related disease are discussed.

## Heterochromatin and cell identity

From a molecular perspective, the differentiated identity is maintained by two parallel mechanisms: one is the machinery that enables the differentiated cell to express its unique gene signature (a topic outside this review), and the second is the machinery that actively silences previous fates and irrelevant gene programs. While the silencing of gene expression also involves post-transcriptional mechanisms, such as RNA decay and protein degradation [19–21], the present review focuses on molecular connections between maintenance of cell identity, heterochromatin formation, and large-scale nuclear organization.

Traditionally, the genome is divided into two entities: euchromatin, which is characterized by an accessible and loose chromatin structure that is highly transcribed and expressed, and heterochromatin (HC), which is dense, compact in structure, and less accessible and, therefore, genes within HC are rarely expressed [22, 23]. HC is divided into constitutive heterochromatin (cHC) and facultative heterochromatin (fHC). HC is spatially distributed in the nucleus, and occupies the nuclear periphery, the vicinity of the nucleolus, and is also situated in and adjacent to centromeres and telomeres and contains repetitive elements and integration sites of foreign elements, e.g., retroviruses. Activation of these genomic entities is harmful to the genome as it may induce recombination and DNA damage, and so, silencing these structures in HC has evolved as a way to make these regions less accessible. HC is also localized to the vicinity of the nucleolus, the largest substructure in the nucleus, containing hundreds of rRNA genes critical for ribosome biogenesis and cell growth. These large chromatin regions are repressive environments and are gene poor, less transcribed, and are highly enriched for satellite DNA and silent genes (for detailed reviews see [24, 25]).

In contrast, fHC has a condensed structure that can change its organization under specific developmental conditions. This flexible form of HC harbors silent genes, and may include regulatory elements, such as lineage and cell-type specific enhancers [26, 27]. A recent study classified a functional type of HC termed sonication-resistant heterochromatin (srHC) that is involved in gene silencing and cell identity ([28] and see detailed below). While all cell types share similar genomic regions of cHC, the identity of fHC and srHC varies for different cell types and developmental settings. In addition, while cHC is associated with the nuclear periphery, fHC can also be found in the nucleus interior [29].

HC is characterized by specific posttranscriptional modifications and, specifically, by histone tail methylations, including histone 3 Lys 9 di- and tri-methylations (H3K9me2/3). These methylations are catalyzed by evolutionarily conserved histone H3K9 methylases (HMT) the SET-domain-containing family of methyltransferases [30]. Once methylated, H3K9me regions are bound by one of the heterochromatin protein1 (HP1α, β, γ) proteins that recognize the methylated histone and self-multimerize and generate inaccessible compact chromatin [31, 32]. Binding of HP1 to methylated histones subsequently recruits repressive complexes or assists in targeting these regions to the nuclear lamina. In some cases, like in neurospora, H3K9 methylation leads to the recruitment and activity of DNA methylases that methylate CpG dinucleotides, linking chromatin silencing with DNA methylation and transcriptional repression [33–37]. Moreover, binding of HP1 to these DNA methylated genes also regulates alternate pre-mRNA splicing [38].

## H3K9me safeguard cell identity

**Fig. 1:**
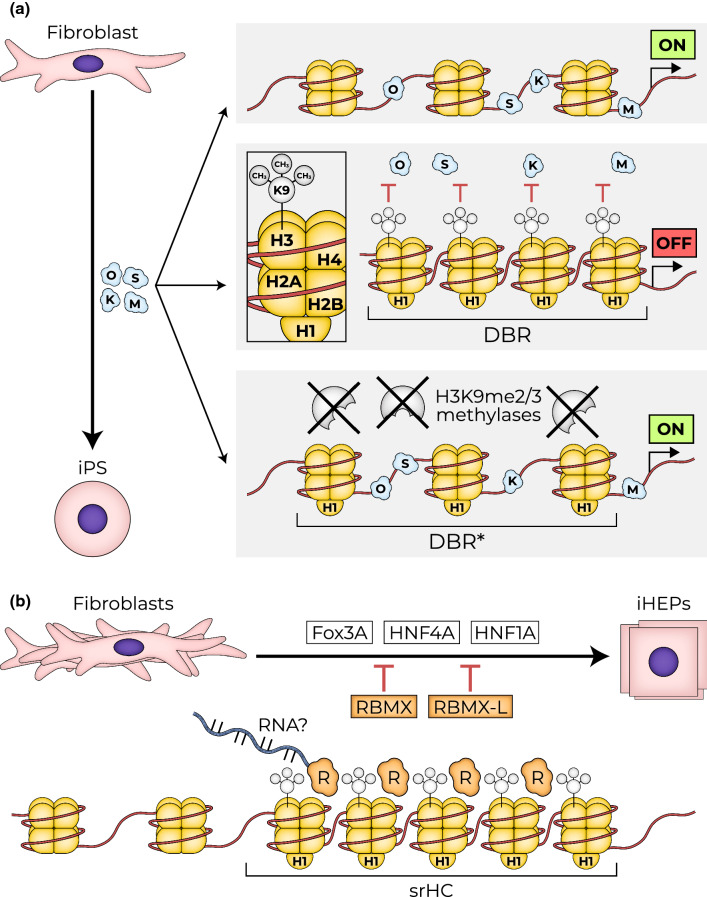
H3K9me2/3-marked heterochromatin attenuates reprograming. **a** Reprogramming of fibroblast to iPs: Expression of Oct4, Sox-2, Klf-4, and c-Myc (OSKM) transcription factors in fibroblast results in generation of iPSs. H3K9me3-marked heterochromatin at developmentally bound genomic regions (DBR) prevent the binding of OSKM to these genomic regions. Eliminating H3K9me2/3 methylases enables OSKM binding to DBR regions (DBR*) and enhances reprograming [40]. **b** Conversion of fibroblasts to hepatocytes (iHEP). Expression of hepatic “founding” transcription factors converts fibroblasts into hepatocytes. iHEP is inhibited by RBMX and RBMX-L RNA-binding proteins. RBMX and RBMX-L were identified as bound to H3K9me3-sonication-resistant heterochromatin (srHC). Their elimination during iHEP generation increased conversion efficiency [28]

Our understanding of the role of H3K9me3 in maintaining the differentiated identity has benefited from experiments of somatic cell reprogramming into iPSs. Reprogramming is an inefficient process, both when achieved by the expression of reprogramming factors (OSKM), and by somatic cell nuclear transfer (SCNT) [28, 39, 40]. This suggests that endogenous barriers that maintain the differentiated state exist and attenuate reprogramming. Indeed, during reprogramming, OSKM binding is not observed along mega-bases of genomic regions decorated with H3K9me3 methylation. These regions, which are termed differentiation bound regions (DBRs) (Fig. 1a) [40], contain numerous genes involved in pluripotency. The inability of reprogramming factors to bind to these regions impedes reprogramming. Reduction of H3K9me3 methylation by knockdown of H3K9me HMTs improved the binding of Oct4 and Sox2 to these regions and enhanced reprograming efficiency. Likewise, and upon differentiation of ES cells during early embryogenesis, hetero-chromatization of pluripotency-related genes like Oct3/4, Nanog, Stella and RX-1 prevents potential de-differentiation [41, 42].

Moreover, during cell specification, marking of chromatin regions by H3K9me3 prevents the expression of irrelevant genes. For example, T helper cells differentiate to either Th1 or Th2 cells, where the expression of Th1-related genes is silenced by H3K9me3 methylation and HP1 binding in Th2 cells. Genetic loss of H3K9me3 methylase (SUv3-9H1) or HP1 resulted in inappropriate expression of Th1 genes in Th2 cells [43]. Similarly, loss of H3K9me3 methylases in the brain resulted in impairment of neuronal identity and electrical activity [44]. In agreement with these reports, double knockout of the H3K9me3 methylases, *suv39h1/2,* resulted in increased chromosomal instability and high risk for tumor development. Extensive loss of H3K9me3 across large chromatin regions has been observed in cancer cell lines and cells undergoing epithelial-to-mesenchymal transition [45–47]. These findings are in accordance with global de-compaction of chromatin observed by pathological optimized imaging (pathSTORM), and likely reflect early events of tumorigenesis [48]. Thus, HC and specifically regions marked by H3K9me3 maintain cell identity and serve as a barrier against spontaneous or experimental reprogramming, as well as tumorigenesis.

## H3K9me and cell fate conversion

Does H3K9me3-marked HC protect against cell fate conversion (trans-differentiation)? A seminal study by Becker et al. sheds light on this question. As a starting point, heterochromatin was isolated based on its biophysical properties, independent of histone-tail modifications [28]. Specifically, when sonicated chromatin is resolved over sucrose gradient by ultracentrifugation, large chromatin fragments that fail to undergo sonication are localized to the middle of the gradient and enriched in DBRs. Fractions containing these regions are not typically collected in regular ChIP-seq assays, where only small fragments from the top of the gradient are used. These short fragments (200–600 bp) are mainly enriched in euchromatin, and specifically in promoter and enhancer regions. Thus, biophysical enrichment of heterochromatic regions enabled isolation of srHC, independent of histone-tail modifications. Remarkably, srHC was greatly enriched in H3K9me3, mapping to DBRs. Moreover, proteomic analysis of the srHC and H3K9me3-enriched chromatin identified bona fide heterochromatic proteins, including several RNA-binding proteins, such as TDP43, that are associated with amyotrophic lateral sclerosis (ALS) and were previously shown to attenuate reprogramming [49].

To directly study the role of srHC/H3K9me-bound proteins in cell conversion, the Zaret laboratory shRNA-eliminated fifty srHC-associated factors upon direct fibroblast conversion to hepatocytes (iHEP; Fig. 1b; [28]). Among the strongest hits of the screen were the RNA-binding proteins RBMX and RMBMX/L. RBMX was previously identified as a regulator required for maintenance and protection of sister chromatid cohesion [50]. It was also required for limiting HIV provirus production by binding downstream to the HIV pro-viral long terminal repeats, maintaining its silencing [51]. Loss of RBMX or RBMX/L proteins alone did not lead to spontaneous cell conversion but rather accelerated fibroblast conversion upon expression of hepatocyte-converting factors (e.g. FOX3A, HNF1A, HNF4A), enhancing the expression of bona fide hepatic genes. Like in the case of iPS reprogramming, loss of SUV39H1 accelerated iHEP conversion. Moreover, RBMX and RMBMX/L1 were required to globally maintain H3K9me3 and, together with SUV39H1, were essential for maintaining srHC. Indeed, the ectopic gene signature emerging for loss of SUV39H1 highly overlapped with that of loss of RBMX/L1 during iHEP. Future studies will be needed to test a possible link between the RNA-binding function of RBMX and RBMX-L, the identity of RNA molecule, and the mechanisms involved.

Using a similar approach, in search for additional genes that attenuate conversion of fibroblasts to iPS or trans-differentiation, Cheloufi et al. identified the histone CAF-1 chaperone complex (ChAF1a, ChAF1b) and the SUMO-conjugating enzyme UBC9 as such factors [52]. CAF1 is a histone chaperone required for replication-dependent nucleosome assembly [53]. It is also involved in heterochromatin maintenance and epigenetic memory, along with the histone de-methylase LSD1 and the SETDB1 H3K9 methyltransferase. CAF-1 elimination decreased HC, resulting in increased accessibility of regulatory regions, such as enhancers, and facilitated binding of Sox2 to genes involved in stemness and promoted their expression. Indeed, CAF-1 limited trans-differentiation of B cells into macrophages or fibroblasts [52]. Moreover, the SUMO pathway was shown to be required to stabilize and protect various cell fates; loss of SUMOylation globally affected chromatin states, including a decrease in H3K9me3-marked heterochromatin [54]. Collectively, these studies demonstrated that both nuclear factors linked to H3K9me-enriched heterochromatin and the SUMO pathway are critical for maintaining cell identity [52, 55, 56].

## Anchoring H3K9me3 heterochromatin to the nuclear periphery

Recent studies in *C. elegans* shed light on the nuclear organization of H3K9me2,3-marked heterochromatin. H3K9 methylations are required In *C. elegans* to anchor and silence repeat-rich heterochromatin at the nuclear periphery. Only double null mutants of *met-2* and *set-25* (DKO), which catalyze the methylations of H3K9, were unable to anchor repeat-containing sequences to the nuclear periphery [57]. Experiments in DKO worms lacking H3K9 methylation resulted in the incorrect positioning of chromosome arms and failure in dosage compensation [58, 59]. Thus, H3K9 methylations have multiple roles in heterochromatin positioning as well as in chromosomal and nuclear organization.

**Fig. 2:**
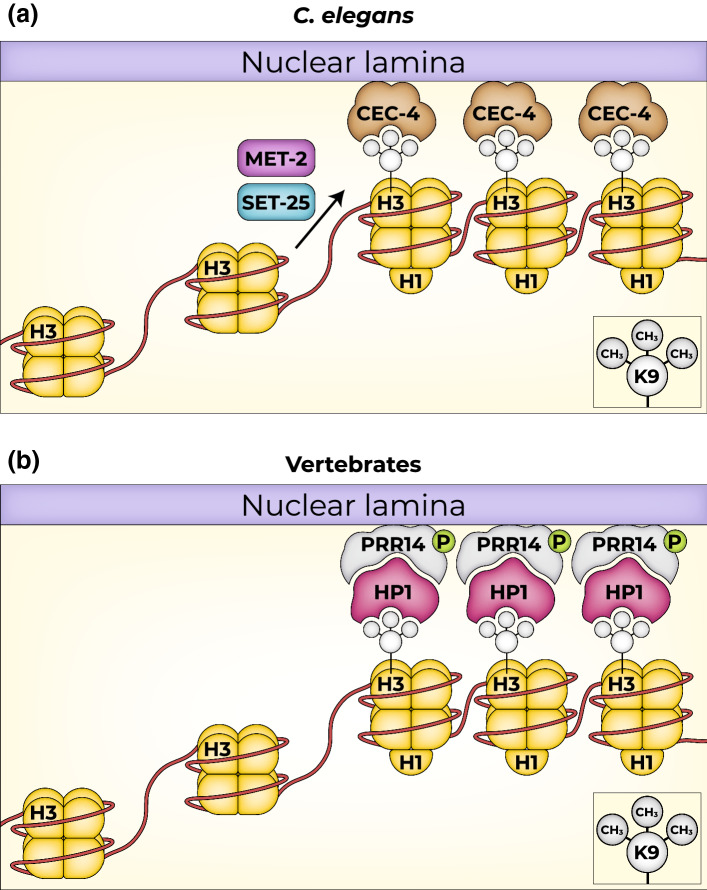
Mechanisms of anchoring heterochromatin to nuclear lamina. **a** In *C. elegans,* histone methylases SET-25 *and* MET-2 di and tri methylate H3K9*.* Subsequently, H3K9me2/3 is anchored to the nuclear lamina by the direct binding of CEC-4, resulting in sequestration of heterochromatin in the nuclear periphery [58]. **b** In vertebrates, H3K9me2/3-marked heterochromatin is bound by HP1. HP1 is recognized and binds to PRR14. PRR14 also has a lamina-binding domain that is required for anchoring H3K9me2/3 heterochromatin to the nuclear lamina, likely in a phosphorylation-dependent manner [85]

The *C. elegans* genome codes for several proteins that are “readers” of H3K9me2, 3 (LIN-61, CEC-3/EAP-1, CEC-4, HPL-1, HPL-2) [60]. Using an in vivo screen to identify proteins that are required to internalize a heterochromatic reporter of engineered repeats, Gonzales-Sandoval identified the chromodomain-containing protein CEC-4 as being required for anchoring of H3K9me-decorated heterochromatin to the nuclear periphery, but not for transcriptional silencing. High-resolution imaging studies suggested that CEC-4 is localized to the vicinity of the nuclear lamina, but independently of the sole *C. elegans* lamin protein (LMN1), and that it binds to mono-, di- and tri-methylated H3K9. When muscle differentiation was forced in the developing larva, the majority of *cec-4* mutants failed to fully commit to muscle fate, likely due to the inability to silence other fate programs (Fig. 2a) [61]. In agreement, Loss of *CEC-4* suppressed the muscle phenotype associated with a point mutation (LMN-Y59C) mimicking human Emery-Dreifuss muscular dystrophy (EDMD) in a *C. elegans* model [62], thus suggesting a connection between the H3K9me heterochromatin-anchoring machinery and lamin.

In line with these observations, Cabianca et al. studied differentiated intestinal cells and discovered a second indirect machinery safeguarding heterochromatin, in addition to CEC-4-dependent anchoring [63]. A screen performed in the background of CEC-4 null mutants identified that MRG-1, the worm ortholog of human MRG15, limits the binding of the CBP/p300 factors, preventing the spread of transcriptional activating histone marks, such as H3K27 acetylation into heterochromatin. Mapping the binding of MRG-1 via ChIP-seq and its comparison with the modENCODE database, revealed that MRG-1 binding is enriched in euchromatic regions marked by H3K36me2/3. Indeed, loss of *met-1* or *mes-4* (the histone methylases that catalyzes H3K36me2/3) in the *cec-4* mutant background resulted in displacement of an artificial repeat reporter from heterochromatin, similar to that observed upon loss of MRG-1.This demonstrates that the positioning of heterochromatin in differentiated cells involves the activity of MRG-1 and H3K36 methylases in euchromatin regions. Interestingly, the shRNA-mediated loss of dMES-4 in differentiated enterocytes (ECs) *in the Drosophila* midgut resulted in a loss of the differentiated identity of mature enterocytes, a decline in the expression of EC genes, and the ectopic expression of the intestinal stem cell marker Delta on the surface of EC-like cells [64].

In humans, NSD2, the ortholog of MES-4, has a tumor-suppressive function and is deleted or mutated in Wolf-Hirschhorn syndrome, which involves a cranio-facial phenotype, intellectual disability, and high incidence of leukemias (T-ALL). NSD2 loss is also characteristic of pediatric B cell lymphomas [65–67]. Taken together, heterochromatin confinement to the nuclear periphery is mediated by multivalent interactions and multiple pathways that together supervise cell identity and prevent tumorigenesis.

## Nuclear periphery, lamins, and cell identity

The formation of repressive chromatin and the regulation of gene expression are tightly connected to the nuclear periphery and to nuclear lamins. Nuclear lamins are type-V intermediate filaments that generate a network in the nucleus, which, in most cases, is dense and associated with heterochromatin below the nuclear membrane, and is sparser in the nucleus interior, where euchromatin dominates [68, 69]. One interesting exception to this organization is observed in rod cells of nocturnal animals, where the organization is inverted and heterochromatin is confined to the nucleus interior [70–72].

Type A lamins (lamin A and C) are encoded by the *LMNA* gene and are generated by alternative splicing. Type B lamins are encoded by the *lamin B1* and *lamin B2* genes. The *C. elegans* genome contains a single lamin gene and the *Drosophila* genome encodes a single type A lamin, termed *lamC*, and a single type B lamin, *lamDm0* [73]. Nuclear lamins interact with a variety of proteins, termed nuclear envelope trans-membrane proteins (NETs) that either the outer or the inner nuclear membranes, which are continuous with the endoplasmic reticulum membrane; their contribution to gene regulation is emerging [74, 75]. Type A lamins are highly expressed in fully differentiated somatic cells, while type B lamins are expressed in almost all cell types. Lamins have transcriptional and non-transcriptional roles that are hard to separate; Lamins greatly contribute to the physical rigidity of the nucleus, their role in gene expression regulation and genome organization is the focus of many studies.

In the nucleus, genomic regions associated with lamins are organized in a distinct manner and form lamin-associated domains (LADs), mainly at the nuclear periphery, where chromatin is in proximity to the nuclear lamina [76–80]. LADs were originally identified by the Van-Steensel laboratory, who used the DamID technique to fuse *Drosophila* LamDm0 to bacterial DNA adenine methylase, which methylates adenine in G^m^ATC sequences [81–84], thus enabling identification of genomic regions in close proximity to lamin. Extensive LAD studies have demonstrated that most LADs are relatively gene-poor, and that genes that present within LADs or are targeted to LADs are less expressed [77, 85]. In addition, it was found that LADs tend to replicate late and are characterized by H3K9me2/3 modifications. LADs are large, ranging from 10 Kb to 10 Mb in size and occupying about one-third of the human genome. Indeed, multiple 3-C, 4-C, and Hi-C chromosome conformation capture studies unveiled that LADs mostly occupy chromosome territories associated with the “inactive” B compartment of the nucleus, and not the “active” A compartment (see glossary and [78]).

Similar to HC, LADs can be categorized as constitutive LADs (cLADs) and facultative LADs (fLADs) [80]. cLADs are highly identical between cell types in both mice and humans, and likely reflect a rigid scaffold anchoring of the genome [86]. In contrast, fLADs are specific for cell type and developmental stage; for example, many genes are found to move in and out of LADs during muscle differentiation, in correlation with their expression [87].

It is not fully clear how chromatin is anchored to LADs. However, the huge size of LADs (encompassing thousands of nucleosomes), together with genetic experiments, suggests the existence of multiple interactions that are only partly dependent on H3K9me [88]. Interestingly, and in reminiscence of CEC-4 in *C. elegans*, the human PRR14 protein that binds HP1 may be involved in anchoring chromatin to LADs in a phosphorylation-dependent mode. PRR14 has separate HP1-binding and lamin-binding domains and likely links H3K9me2/3 HP1-bound and enriched HC to LADs (Fig. 2b) [89]. PRR14 is required for myogenic differentiation of C2C12 myoblasts and for the stability of HP1α laminA/C proteins [90]. However, other lamin interactors, such as lamin B receptor (LBR), which spans the nuclear membrane, likely play a role in tethering HP1-coated-H3K9me chromatin to type B lamins. Indeed, loss of both LBR and laminB1, along with reduced HC-containing LAD were observed in cells undergoing senescence [91]. Moreover, during X-chromosome inactivation, LBR interacts with Xist RNA and is required to localize the X-chromosome to the nuclear lamina [92, 93].

**Fig. 3:**
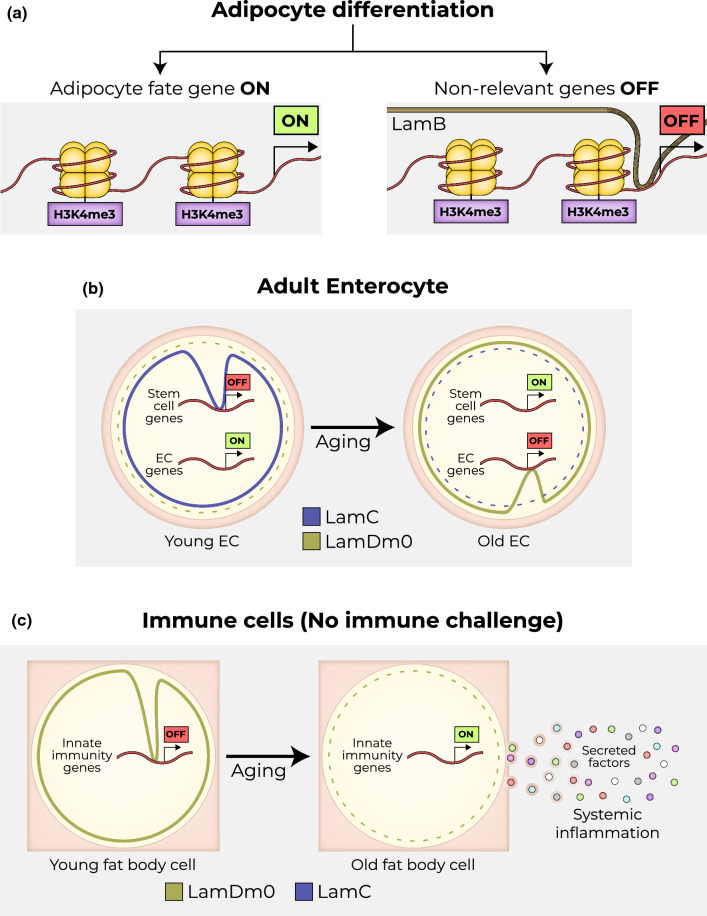
Lamin actively silences gene expression. **a** During adipocyte differentiation, adipocyte fate genes are expressed. Conversely, LaminA/C binds to the vicinity of transcriptional start sites and prevents the expression of other fate genes, albeit the observation that histone tails within the regulatory regions of these genes are marked by activating histone mark, such as H3K4me3. **b** Loss of LamDm0 in fat body cells during aging results in ectopic activation of immune gene signature and in systemic inflammation. **c** Aging ECs flip lamin organization, reverting to a stem-like configuration

**Fig. 4:**
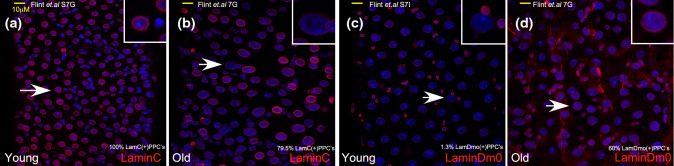
**a**–**d** Expression of lamins in young and old midguts. Confocal microscopy images of young (4 days) and old (4 weeks) adult-derived midguts immuno-stained as indicated. DAPI (blue) marks DNA and arrows points to cells shown in insets (**a**, **b**) in young adults, LamC (red) is homogenously expressed in all ECs, and its level is reduced in aged ECs. **c**, **d** Lamin Dm0 (red), the stem cell-related lamin, is expressed only in progenitor cells in young guts, but is ectopically expressed in polyploid EC-like cells in old guts. The precent of polyploid cells (PPCs) that are positive for the indicated protein in the figure is presented. The figure is adopted from [109]

The regions between LADs, termed inter-LADs (iLADs), are highly transcribed, are associated with active and elongating Pol-II, and are in the vicinity of transcription factories and splicing speckles. The borders of LADs and iLADs are highly enriched with H3K27me3, which is a histone mark associated with fHC and polycomb repression [62, 77]. Early reports suggest that LADs and nuclear pore complexes (NPC), which span the nuclear membrane and are hubs for transcription and DNA repair [94], should be considered separate entities Figs. 3 and 4.

A recent study in *Drosophila* cells identified significant binding (~ 20%) of two NPC proteins Nup93 and Nup107 within LADs [95]. Nup93-bound regions were associated with polycomb-repressed chromatin regions and LADs. Moreover, in some cases, the peak binding of Nup93 within LADs was correlated with low signal of lamin-binding, suggesting a unique chromatin environment within the LAD itself. Additional studies will be necessary to further understand this observation, and to determine whether this association of Nup subunits to LAD sub-regions reflects binding of isolated subunits or entire NPCs.

## NET proteins and tissue-specific anchoring of chromatin to the nuclear periphery

The above observations suggest a general role for H3K9 methylations and lamins in the tethering of genomic regions to the nuclear periphery. Elimination of all lamins from mouse embryonic stem cells revealed, however, that they are dispensable for lamina-associated domain organization in cells [96]. While this may be relevant only in the case of embryonic mouse stem cells, it strongly suggests that chromatin is tethered to the nuclear periphery by proteins other than lamins.

Among tissue-specific anchoring proteins, there are nuclear envelope proteins associated with inner nuclear membrane (INM), as well as transmembrane proteins collectively termed NET (for reviews on NET, see Wong et al. [74] and Talamas and Capelson [97]). NET proteins are involved in many cellular functions including nuclear migration, signaling, cell cycle regulation, and genome organization [98]. Some NET proteins, such as LEM-domain proteins, are localized only to the INM. Other INM NET proteins, such as SUN-domain proteins, form a complex with Nesprin proteins that reside in the outer nuclear member (ONM), and together form a complex, termed LINC, that conveys cytoplasmic signals and mechanosensory information to the nucleus.

Remarkably, proteomic analyses expanded our view on NET proteins by identifying hundreds of novel NET proteins. A comparison of these proteomic studies performed on different cell types revealed that the NET proteome is highly cell/tissue-specific. This cell/tissue-specific expression is important, for example, for selective docking of chromosomes near the nuclear periphery. In liver cells, NET proteins NET29, 39, 45, and 47, but not other NET proteins tested, were able to reposition chromosome 5 to the nuclear periphery. In contrast, kidney cells do not express NET47 and in the majority of these cells, chromosome 5 is localized more to the nuclear interior. Similarly, NET 29 and 39, but not others NET proteins, were required for repositioning of chromosome 13 [99, 100].

Other examples of NET proteins tethering the genome to the nuclear periphery are the LEM-domain proteins, Emerin and LAP2 β [101]. Localized to the inner nuclear membrane, they contact the genome indirectly by binding to the bridging protein BAF that binds to chromatin. For example, LAP2β prevents the expression of cardiac genes maintaining the identity of cardiac progenitors [102]. In muscle progenitor cells, Emerin tethers differentiated genes to the nuclear periphery, maintaining stemness [103, 104]. Moreover, high-resolution DamID mapping, combined with transcriptional analysis during myogenic differentiation, revealed that gene repositioning was regulated by muscle-specific NET proteins, impacting the expression of a large number of developmental genes during myogenesis. Importantly, the cell-specific expression of NET proteins may explain why pathologies associated with lamins or NET proteins are only manifested in specific cells and tissues.

## Lamins in the nuclear interior and cell identity

DamID and ChIP-seq-based LAD mapping of LaminB and LaminA/C yielded highly similar results [105], with both localizing mostly to peripheral LADs. Recent studies, however, suggest a repressive function for lamins in the nucleus interior, specifically LamA/C [29]. For example, in the case of adipocyte differentiation, the binding of lamin A/C was sufficient to prevent the expression of genes with histone marks associated with gene activation (e.g., H3K4me) [106].

Moreover, during in vitro adipocyte differentiation, LamA/C formed a unique type of LAD that is associated with H2B-acetylgluscosamine-enriched chromatin (H2B112GlcNAC), suggesting a chromatin-related nutrient-sensing machinery during differentiation [107].

The organization of LamA/C in the nucleus interior is likely different from its tight peripheral association with the nuclear lamina. In the nucleus interior, it is observed microscopically like a “veil” and is easily extracted [29]. This may be partly due to the association of LamA/C with LAP2α (thymopetin, TMPO) an abundant nuclear protein, as loss of LAP2α resulted in depletion of LamA/C from euchromatin [108].

## Regulation of the differentiated identity is sequentially molded by lamin networks

In many somatic cells, both types of lamins are co-expressed, with each generating distinct networks that do not mix [68]. During mouse embryogenesis, however, a lamin-B receptor (LBR) network tethers HC to the nuclear periphery, preventing premature differentiation. Subsequently, and during differentiation, this network is replaced by a LamA/C-dependent tethering mechanism that enhances differentiation, preventing expression of stem cell genes. Loss of both LBR and LamA/C results in inverted organization in which HC is localized in the nucleus interior [70, 80].

The active role of lamins in supervising the identity of stem cells and differentiated cells was studied in several *Drosophila* tissues. For example, loss of LamDm0 in female-derived fat body cells, led to detachment of testis (male)-specific gene clusters from the nuclear lamina and to their ectopic expression [109]. The active and sequential role of lamins in the regulation of cell identity was also studied in *Drosophila* enterocytes (ECs) [110]. The distribution of lamins in the *Drosophila* midgut is differential; in intestinal stem cells (ISCs), the dominant lamin is the B-type lamin, LamDm0. Upon differentiation, LamDm0 levels decline and the level of LamC increases and is the dominant lamin in ECs. The switch in *lamin* gene expression is directly regulated by the HES-related transcription factor Hey, which binds to enhancers in the lamin genes, repressing the expression of stem-cell lamin, LamDm0, and enhancing the expression of the differentiated LamC [110]. Genetic experiments, together with DamID profiling, established that in stem cells, LamDm0 binds to hundreds of EC genes, preventing their expression and maintaining stemness. Loss of LamDm0 in ISCs resulted in ectopic expression of EC genes, e.g., the EC founder transcription factor Pdm1. Moreover, forced expression of LamDm0 in ECs suppressed the expression of the entire EC gene program. Likewise, in differentiated ECs, LamC silenced the expression of stem cell-related and irrelevant gene programs, and its elimination in ECs resulted in ectopic expression of stem cell genes, such as the Notch receptor Delta. Thus, each lamin actively shapes a unique nuclear organization, preventing the expression of specific gene programs. It remains, however, to be determined how the different sets of genes are distinguished in each cell, and the molecular mechanism linking each lamin to its repressed targets also remains to be identified. Remarkably, loss of identity in ECs due to the elimination of either Hey or LamC, has an impact on the entire tissue, including a pathological regenerative response of stem cells, mis-differentiation, loss of tissue integrity, and reduced organismal viability. Thus, a Hey-lamin network establishes and supervises EC identity.

## Supervising cell identity in the context of aging

Aging is intimately linked to loss of cell identity and is associated with age-related diseases, including increased susceptibility to infection [111]. It is characterized by a plethora of cellular changes, including aberrant signaling, such as in the mTOR pathway, mitochondrial dysfunction, and rewiring of metabolic networks with effects on both the tissue and the organism level.

In the nucleus, aging is associated with changes in the epigenome, the function of nuclear pores, and large-scale re-organization of the nucleus affecting nuclear lamins and intranuclear organelles [112, 113]. These changes can be envisioned as a lowering of the height differences between the valleys and hills depicted by Waddington. Transcriptionally, it is reflected in the reduced expression of cell-specific programs, loss of silencing, and increase in transcriptional noise [114, 115]. For example, genome-wide mapping established that open chromatin regions that are active in dividing young cells, become increasingly/more closed upon aging, while regions of compact HC become more accessible.

These changes are likely due to a decline in the activity and levels of identity supervisors, leading to aging chromatin that is more homogeneous [116]. Among the changes observed are relaxation of cHC and the ectopic expression of transposable elements (TEs) that has the potential to induce DNA damage [117, 118]. The ability to silence TEs has been linked to the function of lamin in *Drosophil*a, where depletion of LamDm0 from the fat body of young adult larvae resulted in decreased HC levels and re-expression of TEs [119]. Similarly, laminA/C are required for silencing LINE-1 TE in vertebrate cells [120]. Moreover, maintaining cHC structure requires interaction of lamins with nuclear-cytoskeleton organizers that together shape a cell-specific nuclear state. One such organizer is Washout (wash), a member of the Wiskott–Aldrich syndrome family of proteins, that are well-known to regulate cytoplasmic signaling, as well as membrane–cytoskeletal interactions, including the formation of branch-actin filaments [121, 122]. Nuclear Wash is required for large-scale nuclear organization; *wash-*deficient *Drosophila* cells exhibit a wrinkled nuclear morphology and disruptions of intranuclear organelles similar to those observed in laminopathic cells. Wash interacts directly with the type B lamin, LamDm0, and is required for cHC integrity. Loss of *wash* results in increased chromatin accessibility and changes in the distribution of repressive histone marks.

How HC is maintained in the context of aging is the subject of extensive studies. At the level of the HC-associated histone tail modification, normal aging correlates with a reduction in H3K9me levels in HC, increase in H3K9me3 outside HC and redistribution HP1 [123]. Studies of accelerated aging syndromes, such as HGPS (a mutation in LamA gene) and Warner syndrome, suggest mechanistic explanations for the loss of HC in aging stem cells [124, 125].

For example, differentiating mesenchymal stem cells (MSCs) derived from Werner syndrome (WRN) patients, a pre-mature aging syndrome, exhibit epigenomic aging phenotypes [126], including global reduction in H3K9me3 levels, changes in chromatin architecture, and premature cellular senescence [127]. WRN helicase, which is mutated or silenced in WRN syndrome, directly interacts with the H3K9 methylase SUV39H1, HP1, and the LamA/C-binding protein LAP2β. In aging cells, reduction of SUV39H1 levels is observed together with the appearance of a WRN-like chromatin landscape. Replacing SUV39H1 with a catalytically inactive SUV39H1 in wildtype MSCs, mimicked WRN phenotypes. Thus, WRN helicase is a regulator of cell identity that protects from premature aging, in part by regulation of H3K9me3-associated HC via regulation of a lamin-associated protein.

The active role of lamins in supervising cell identity in the context of aging was recently investigated in both *Drosophila* adult midgut and immune tissues. In midgut aged enterocytes, the level of Hey protein decreases, leading to a decline in the level of LamC and, as a result, to the ectopic expression of stem cell genes, including LamDm0, and to subsequent silencing of the EC signature. Indeed, the phenotype observed upon acute loss of Hey in young ECs is highly similar to that observed in aged ECs. Remarkably, expression of Hey, or to a limited extent, of LamC, in aged ECs restores lamin organization and suppressed aging phenotypes [110, 128].

Another characteristic of aging is immune senescence, which is associated with activation of immune responses in the absence of a pathogenic challenge [129]. In aging *Drosophila*, factors secreted from the fat body (homolog of vertebrate liver) elicit systemic inflammation, as well as hyperplasia in the midgut [130, 131]. In young adults, the ability to prevent such aberrant activation of immune genes requires LamDm0 within fat body cells. During aging, LamDm0 levels decline, resulting in a decrease in H3K9me-marked HC and HP1 levels, and in ectopic expression of immune-related genes, including secreted factors that mediate systemic inflammation. Along these lines, an age-dependent decrease in LamB1 levels was observed in keratinocytes, as well thymic epithelial human cells [132, 133].

The regulation of cell identity by lamins is also conserved in humans. The ability of cells to divide in vitro is limited by a cellular aging process called replicative senescence. Replicative senescence is associated with changes in chromatin organization, cell cycle arrest, and an increase in metabolic activity and cytokine production [134].

Remarkably, loss of lamin B1 in proliferating fibroblasts induced cellular senescence and was accompanied by changes that are highly similar to cells undergoing senescence, including large-scale changes in the chromatin landscape and in gene expression [135–137]. Interestingly, over-expression of lamin B1 also induced cellular senescence, suggesting that a delicate balance of LamB1 levels is critical for maintaining cell identity [138]. Thus, lamins are required to actively maintain cellular identity, and their decline with age alongside dis-regulation of lamin expression have systemic and organismal manifestations beyond the differentiated cell itself.

Numerous mutations in lamin genes and nuclear lamina proteins are associated with a group of diseases collectively termed laminopathies, which affect the muscular, skeletal, adipose, and neuronal tissues, as well as the heart and skin [139, 140]. A prominent example is a single-nucleotide mutation in the *lamin A* gene (C1824T) that leads to a splice variant of lamin A, which is permanently farnesylated, generating a protein called Progerin, which is the cause of the Hutchinson–Gilford progeria (HGPS), a premature aging syndrome [141, 142].

Interestingly, this single-nucleotide mutation and the *progerin* splice variant are also sporadically present in physiological wild-type aged cells [124, 143, 144].

Analyzing the changes in histone tail modification patterns in HGPS fibroblasts revealed global and rapid reduction of H3K9me3, specifically in the vicinity of the nuclear lamina, along with a decrease in HP1 that binds to H3K9me3 [125, 145]. Moreover, these changes were also observed in HeLa cancer cells upon expression of Progerin. Taking advantage of iPS technology, the Izpisue laboratory recapitulated the nuclear defects, including lamina disorganization and HC loss [145]. This study established that HGPS-derived iPSs do not express Progerin and are indistinguishable from iPSs derived from control fibroblasts in all that pertains to epigenetic, nuclear lamina organization, and proliferation parameters analyzed. However, upon several passages, these cells exhibit nuclear disorganization and a decrease in H3K9me3, as observed in HGPS fibroblast. Moreover, differentiation of HGPS-iPSCs to smooth muscle cells leads to premature senescence phenotypes that are observed in aged smooth muscle and vascular endothelial cells. In addition, using gain- and loss-of-function experiments were used to establish the central role of Progerin in these premature aging-related phenotypes.

These data experimentally substantiated the idea introduced by Goetzman and Foisner that laminopathies originate at the level of somatic stem cells, such as MSCs, which give rise to bone, muscle and cartilage, but are manifested upon differentiation and over time [146]. Indeed, focusing on adult stem cells, Scafidi and Misteli observed that immortalized human Progerin-expressing mesanchimal stem cells change their cellular identity and differentiation potential, in part by activating the Notch/HES pathway and remodeling HC, including HP1γ and nuclear lamins [147].

Ikagami et al. [148] recently observed that in fibroblasts, Ser22-phosphorylated lamin A/C in the nuclear interior was required for the binding of lamin to enhancers that were also co-bound by c-Jun. Remarkably, in Progeria-derived fibroblasts the binding of p-Ser22-LamA/C was reduced at these sites. Moreover, ectopic p-Ser22-LamA/C binding, c-Jun recruitment, and gene activation near Progeria-related genes was observed [148].

Along this line, the Foisner group generated an endothelium-specific HGPS mouse model with selective endothelial Progerin expression. These transgenic mice exhibited deregulated activity of a major cardiac transcription factor termed mechanoresponsive myocardin-related transcription factor-A (MRTFA) and developed myocardial and perivascular fibrosis, left ventricular hypertrophy, and premature death [149].

De-regulated and elevated levels of inflammatory markers were observed in a progeria mouse model [150], whose pathological consequences were recently shown to be ameliorated by genomic editing that reduced the level of Progerin [151, 152]. At the gene-expression level as well as phenotypically, HGPS patients exhibit upregulation of NF-κB activity and elevated level of cytokines. HGPS patients partially benefited from treatment with farnesylation inhibitors [17, 153, 154].

## Biophysics of cell identity

Molecular mechanisms were described that regulate HC-dependent silencing involved in the regulation of cell identity. Chromatin partitioning is, however, also determined by the self-assembly of proteins, the biophysical properties of macro-molecules, such as viscous chromatin, and the nucleoplasm environment. These forces determine intra-nuclear domains, nuclear bodies, and the segregation of chromatin loops, including euchromatin and HC. They govern gene regulatory regions, such as super-enhancers, and accessibility of the transcriptional machinery and repressive complexes [155–160]. For example, Hi-C studies, together with microscopy and polymer simulation, provided evidence for the self-organization of HC in both conventional somatic cells and in rod cells, in which the organization of HC is inverted [71].

At the heart of this “self-organization” phenomenon is a process termed phase-separation and formation of localized condensates [161]. One type of separation is liquid–liquid phase separation (LLPS), which generates liquid droplets that are formed when a homogeneous solution de-mixes into separate phases, generating membranelles, which are organelle-like regions that sequester and concentrate proteins and nucleic acids. In many cases, LLPS is initiated by the focal concentration of proteins containing intrinsically disordered regions (IDR), [162, 163]. In this regard, and highly related to cell identity, HP1 was demonstrated to mediate LLPS and HC [157, 164, 165], and reviewed in [166]. It appears that the N-terminal hinge domains of HP1A and HP1α harbor IDR regions, enabling LLPS and the formation of HP1 droplets. Both studies suggest that the local binding of HP1 molecules to H3k9me3, inoculates a seed that leads to droplet formation in a process that is still not fully understood. Moreover, the binding of the yeast ortholog of HP1, Swi6, to nucleosomes induces conformational changes, resulting in the formation of distorted nucleosomes with aberrant conformation, enhancing phase separation in these regions [166]. HP1 droplets tend to fuse, enabling the entry of nucleosomal DNA but not of the transcription factor TFIIB into these droplets, likely preventing active transcription over extensive genomic regions.

While the above explanations for the establishment and spreading of HP1-dependent HC are attractive, this view was recently challenged [167]. Erdel et al. found that HP1 only weakly forms liquid droplets in mouse fibroblasts. They suggest an alternative view according to which HC compaction can switch between two binary states depending on the presence of a transcriptional activator, without involving HP1 droplets and LLPS. Thus, these differences likely indicate that we are in the early days in which the biophysics and biology of HP1 meet.

Another recent example of LLPS-dependent transcriptional repression involves the developmental co-repressor Groucho (Gro)/TLE 142 [168]. Gro in *Drosophila* and *Ciona*, and TLE/Grg1-4 in vertebrates, are co-repressors that interact with multiple transcription factors, including Hey and other HES-related TFs. Gro/TLE regulates “transcriptional memory” cell fate and identity [169, 170]. Groucho/TLE proteins contain a large IDR region, and to impose repression, they oligomerize and generate a compact chromatin structure [171]. Treen et al. discovered that Gro generates a droplet-exclusion mechanism through its WD40 domain and LLPS, establishing transcriptional repression during embryogenesis. These droplets are likely involved in the formation of compact chromatin and hinder access of transcriptional activating factors from gene regulatory regions. It is interesting to note that Gro-mediated repression is dependent and enhanced by its SUMOylation [172]. Thus, it would be interesting to investigate the contribution of SUMOylation to Gro-dependent LLPS.

Collectively, these pioneering studies suggest that, once determined by genetic factors, the biophysical properties of these proteins and the local nuclear environment self-generate chromatin and nuclear compartmentalization that safeguard cell identity.

## Future directions

As postulated by Blau and Baltimore [12], regulation of cell identity requires continuous supervision. The development of powerful genomic and imaging techniques has enabled researchers to better understand the genomic and large-scale organization of chromatin and nuclear structure in 3D. Likewise, the roles of long non-coding RNA in gene regulation, chromatin organization, and cell identity are emerging [173]. Biophysical studies have highlighted the importance of the intrinsic properties of proteins, chromatin, and the nucleoplasm in the formation of intranuclear compartments. The current challenge is to understand how these layers of regulation are interconnected and integrated into cell identity control. It is important to consider that regulation of cell identity is dynamic, as the differentiated cells must adjust to their ever-changing environment. Thus, molecular sensors that can relay these changes to the nucleus and regulate the activity of identity supervisors likely exist and remain to be discovered. These sensors will likely include genes involved in post-transcriptional modifications. Indeed, lamins are subjected to posttranscriptional modifications, such as phosphorylation, ubiquitination, and SUMOylation, which likely affect their function and stability [174]. In this regard, the power of genome editing and functional screens will help discover critical nodes of regulation.

Finally, observations that epigenomic aging pathways are conserved and that aging phenotypes can be suppressed by re-expression of identity supervisors, are promising [175–177]. Indeed, a recent study demonstrated that the mortality rates of HGPS patients decreased upon treatment with farnesylation inhibitors [178]. One can, therefore, envision that small molecules and drugs protecting the epigenome and nuclear organization will enable attenuation of aging, reduce aging-related diseases, and significantly improve quality of life in aging individuals [112, 179].

## Glossary

A and B compartments in the nucleus: On a large-scale, chromosomes, are spatially organized in the nucleus in relatively fixed chromosomal territories. Within chromosomes, two domains can be observed: The A compartment, which is associated with “active” gene transcription, and the “inactive” B compartment. The separation to A and B compartment is based on Hi-C studies. Regions within each compartment self-associate and tend to have multiple contacts. These regions are multi-Mb size; the A compartment is rich in genes and CG, enriched by histone marks associated with gene activation, and tend to localize to the nucleus interior, while the B compartment tends to be poor in genes, rich in LADs, and localized to the nuclear periphery [180].
Chromosome capture analysis: Chromosome capture analysis (3-5C and Hi-C) comprises two techniques that enable to map organization of chromatin and interactions between genomic regions. They enable to measure interactions between linearly distant regions, offering a visualization of the 3D organization of the nucleus [181].
Euchromatin: A loose structure of chromatin that is more accessible, gene-rich, and active in transcription, and is located mostly in the nucleus interior.
Heterochromatin (HC): A compact and dense structure of chromatin that is less accessible for transcription, has lower activity, and is localized to the nuclear periphery.
cHC: Constitutive heterochromatin is a form of HC that is located mainly in centromeric and telomeric regions. It contains highly repetitive sequences and is transcriptionally inactive.
fHC: Facultative heterochromatin is a flexible form of HC that can be organized differently under specific cell conditions including development context.
srHC: A functional type of HC that is significantly enriched in H3K9me3 and is transcriptionally repressed. Its identity varies in different cell types.
LAD: Lamina associated domains are domains in which the nuclear lamina is associated with the chromatin. LADs are gene poor or harbor less-expressed genes.
cLAD: Constitutive LADs have a conserved genomic position from mouse to human in the different cell types.
fLAD: Facultative LADs are less compact LADs, richer in genes and are cell-type specific.
iLAD: Inter-LADs are regions between LADs that are highly transcribed and positioned away from the nuclear lamina.
